# Different mechanisms of magnitude and spatial representation for tactile and auditory modalities

**DOI:** 10.1007/s00221-021-06196-4

**Published:** 2021-08-20

**Authors:** Alice Bollini, Davide Esposito, Claudio Campus, Monica Gori

**Affiliations:** 1 Unit for Visually Impaired People, Istituto Italiano di Tecnologia, Genoa, Italy; 2 DIBRIS, Università di Genova, Genoa, Italy

**Keywords:** Magnitude, Spatial representation, Frame of reference, Touch, Audition

## Abstract

The human brain creates an external world representation based on magnitude judgments by estimating distance, numerosity, or size. The magnitude and spatial representation are hypothesized to rely on common mechanisms shared by different sensory modalities. We explored the relationship between magnitude and spatial representation using two different sensory systems. We hypothesize that the interaction between space and magnitude is combined differently depending on sensory modalities. Furthermore, we aimed to understand the role of the spatial reference frame in magnitude representation. We used stimulus–response compatibility (SRC) to investigate these processes assuming that performance is improved if stimulus and response share common features. We designed an auditory and tactile SRC task with conflicting spatial and magnitude mapping. Our results showed that sensory modality modulates the relationship between space and magnitude. A larger effect of magnitude over spatial congruency occurred in a tactile task. However, magnitude and space showed similar weight in the auditory task, with neither spatial congruency nor magnitude congruency having a significant effect. Moreover, we observed that the spatial frame activated during tasks was elicited by the sensory inputs. The participants' performance was reversed in the tactile task between uncrossed and crossed hands posture, suggesting an internal coordinate system. In contrast, crossing the hands did not alter performance (i.e., using an allocentric frame of reference). Overall, these results suggest that space and magnitude interaction differ in auditory and tactile modalities, supporting the idea that these sensory modalities use different magnitude and spatial representation mechanisms.

**Supplementary Information:**

The online version contains supplementary material available at 10.1007/s00221-021-06196-4.

## Introduction

Magnitude estimation is the capacity to extract numerosity, luminance, or intensity of environmental features. Knowledge about an object's magnitude allows for accurate estimation of its size or spatial distance, allowing an appropriate selection of the suitable action for interacting with the object. However, this estimation is not a simple task. Indeed, action planning must consider environmental inputs coming from different sensory modalities and knowledge about the body's state and then associate these pieces of information with information about the spatial coordinates of surrounding objects and their magnitude. A relationship between magnitude and spatial representation is required to solve this complex task. This relationship becomes evident in the experimental tasks where participants are asked to discriminate quantities. In this type of task, participants respond faster to smaller quantities when using their left hand, whereas they respond faster to bigger quantities with their right hand (Dehaene et al. 1993). This effect reflects that the mental representation of magnitude is spatially encoded as line-oriented from left to right. Research on the link between magnitude and spatial representation is growing and suggests overlap in the processing of magnitude, temporal, and spatial information. In particular, neuroimaging studies indicate that the parietal regions of the brain are involved in this overlap (Bueti and Walsh 2009; Cantlon et al. 2009; Cona et al. 2021).

Stimulus–response compatibility (SRC) tasks are widely used to investigate the relationship between magnitude and spatial representation. In an SRC task, participants show better behavioral performance (speed and accuracy) when the stimulus (sensory input) and the response (action) share common features (e.g., location or affordance, the proper of an object that defines its possible uses), even if they are task-irrelevant features. Among the classical paradigms used to investigate SRC, there is the Simon task (Simon and Small 1969). In this task, performance is better when the stimulus appears in the same spatial location as the response button. The interesting aspect of the Simon effect is that subjects must respond to a non-spatial feature of the stimulus, like color or sound, and therefore spatial position (left or right) is irrelevant. For example, the instruction may be: “press the right key for green stimuli and the left key for red stimuli”. Participants are faster and more accurate when the stimulus on the screen appears on the same side of the button to be pushed (spatially congruent) than when the stimulus is on the opposite side (spatially incongruent). SRC effects also occur with representational space as opposed to explicit spatial locations. In this case, the irrelevant spatial information is implicit, as in the spatial–numerical association of response codes (SNARC) effect (Dehaene et al. 1990, 1993). Specifically, smaller numbers are associated with the left-hand space, and larger numbers are associated with the right-hand space. This effect has been taken as evidence for the existence of a mental number line (MNL) (Restle 1970); in Western culture, this reflects a spatial continuum from left (small numbers) to the right (bigger number) (Dehaene 1992). In this context, the SNARC effect results from correspondence between MNL representation and action execution (Prpic et al. 2016).

In addition to number representation, a relationship between spatial representation and magnitude can occur, i.e., the mental representation of a countable and uncountable quantity (Gallistel and Gelman 2000). Specifically, SRC mapping has been observed in many non-spatial domains, such as size (Ren et al. 2011; Wühr and Seegelke 2018), brightness (Fumarola et al. 2014), time (Ishihara et al. 2008), and even in highly abstract domains like emotional intensity or risk judgment (Holmes et al. 2019; Macnamara et al. 2018). For example, people are faster to respond to low-risk perceptions or negative emotions when they are in the left space and vice-versa, even though a specific reference for the emotional intensity domain is quite debated in the field (see Baldassi et al. 2021; Fantoni et al. 2019 for an alternative approach to this topic). These studies suggest that left-to-right representation effects exist in other sensory domains besides vision. For example, in the auditory modality, several sound features show the automatic mapping of magnitude in space (Bruzzi et al. 2017; Fairhurst and Deroy 2017; Hartmann and Mast 2017; Lidji et al. 2007; Rusconi et al. 2006; Weis et al. 2016). In particular, the effect on the auditory domain, known as SMARC (Spatial Musical Association Response Code; Rusconi et al. 2006) or SPARC (Spatial Pitch Association Response Code; Lidji et al. 2007) has been demonstrated using pitch as the auditory stimulus. Pitch is determined by the sound frequency (Moore 2003) in many languages and can be defined as high or low according to the magnitude of its frequency. This language preference has led researchers to hypothesize that the SPARC effect occurs for vertically oriented response mapping. Indeed, it has been demonstrated that this effect is present on the vertical axis and the horizontal axis in the case of expert musicians (Lidji et al. 2007; Rusconi et al. 2006). Indeed, expert musicians might develop a mental pitch line that is horizontally distributed according to their experience with a musical instrument, like the piano keyboard, where musical notes are left-to-right mapped. A recent study has suggested that this representation might be due to the visual representation of musical notes on the stave (formally trained musicians are used to read music when playing) rather than on the layout of the specific musical instrument played (Fumarola et al. 2020). Either way, recent studies have also found a horizontally distributed SPARC effect in non-musicians (Fischer et al. 2013; Weis et al. 2016). Moreover, SNARC-like effects are also seen in the tactile domain (Bollini et al. 2020; Brozzoli et al. 2008; Krause et al. 2013). While Brozzoli et al. (2008) and Krause et al. (2013) investigated the number representation of finger counting, in our previous work, we investigated the role of magnitude in the tactile modality (Bollini et al. 2020). We demonstrated with an SRC task in the tactile modality that the effect of magnitude congruency was more substantial than that of spatial congruency. The presence of SRC effects across different sensory modalities supports the existence of a universal representation of magnitude (Walsh 2003). This magnitude system is now known to be located in the parietal cortex (Bueti and Walsh 2009) and to process all sensory information about space, time, and numbers. However, the role of sensory information in magnitude processing has not been fully investigated. Specifically, it remains unclear whether all sensory systems process magnitude in the same way or whether they differ in how they weight the representation of magnitude.

A strong association between space and magnitude exists, suggesting the presence of a reference frame (Gevers and Lammertyn 2005). Notwithstanding, a clear understanding of the reference frame characterizing the relationship between space and magnitude is missing (Viarouge et al. 2014). In particular, it remains unclear whether spatial-magnitude effects (e.g., SNARC-like effects) are represented in an egocentric frame (i.e., body-centered) or an allocentric frame (i.e., object-centered). A recent study proposed that the spatial frame activated for magnitude depends on the demands of the experiment (Viarouge et al. 2014). This idea implies that the spatial frame may depend on the specific task's sensory modality. In agreement with this idea, it has been shown that different sensory modalities can influence the spatial reference frame in an SRC task (Ruzzoli and Soto-Faraco 2017). One way to test the spatial reference frame in SRC and SNARC-like effect tasks is to compare the participants’ performance between uncrossed and crossed hands. In this way, the experiment introduces a misalignment of egocentric and allocentric frames of reference for coding the side of the stimuli when the arms are crossed over body midline (the right hands is located in the left hemispaces and vice versa) (Dehaene et al. 1993; Roder et al. 2007; Schicke and Rôder 2006; Shore et al. 2002; Wood et al. 2006).

To date, it remains unclear how sensory inputs affect the relationship between space and magnitude. We hypothesized that magnitude congruency effects would differ for auditory and tactile tasks, as the reference frame differs between the two modalities, with auditory tasks relying more on allocentric frames while tactile tasks relying on egocentric frames (for complete reviews, see Badde and Heed 2016; Voss 2016).

To test our hypothesis, we investigated the role of auditory and tactile information in different conditions of an SRC task. First, we created conflictual spatial and magnitude mapping codes to disentangle the roles of magnitude and spatial representation. Second, to understand the role of the spatial reference frame in magnitude congruency effects, we manipulated the hands' position. Namely, participants were required to respond with their hands crossed over the body midline, thus, creating a conflict between egocentric and allocentric representations.

## Methods

### Participants

Forty participants were enrolled in this study. All signed written informed consent forms following the Declaration of Helsinki. The ethics committee approved the local health service study (Comitato Etico, ASL3 Genovese, Italy). None of the participants has a formal musical education. Data from two subjects were excluded because, in one case, the accuracy was under 50%, and in the other technical issues arose during the experimental session. The sample size was calculated based on our previous study (Bollini et al. 2020).

### Apparatus and stimuli

The experiment was run using Psychtoolbox-3 (Kleiner et al. 2007) on Matlab® 2018b. Tactile stimuli were the same used in previous work (Bollini et al. 2020): vibrotactile stimulations delivered through two modules of MSI Caterpillar (Gori et al. 2019) placed on participants' right and left wrists. The tactile target stimuli were two 100 ms vibrations (stimulus 1: 60 Hz 2 V; stimulus 2: 120 Hz 3 V) that could appear on either the left or right wrist. Before the target stimuli, we gave the warning to advise the participant a stimulus was coming. The tactile warning was a vibration with an intermediate amplitude and frequency between the two target stimuli that were presented simultaneously to both wrists. Two speakers were placed 60 cm from the participants for the audio stimuli and separated by 120 cm. The auditory target stimuli were two pink noise bursts lasting 100 ms (stimulus 1: 500–3000 Hz 80 dB; stimulus 2: 500–15,000 Hz 81 dB) presented in either the right-hand or left-hand speaker; the speakers were at ± 90° from participants' body midline. The warning tone was a sine-wave tone lasting 250 ms (1000 Hz 87 dB) presented simultaneously to both speakers. Stimuli were chosen based on a pilot study (*n* = 10) and designed to equalize difficulty in the discrimination of audio and tactile stimuli and have an overall accuracy level of around 85%. Responses were collected using a push-button panel placed in front of participants and 14 cm away from the body midline in the left and right hemispaces.

### Procedure

**Fig. 1:**
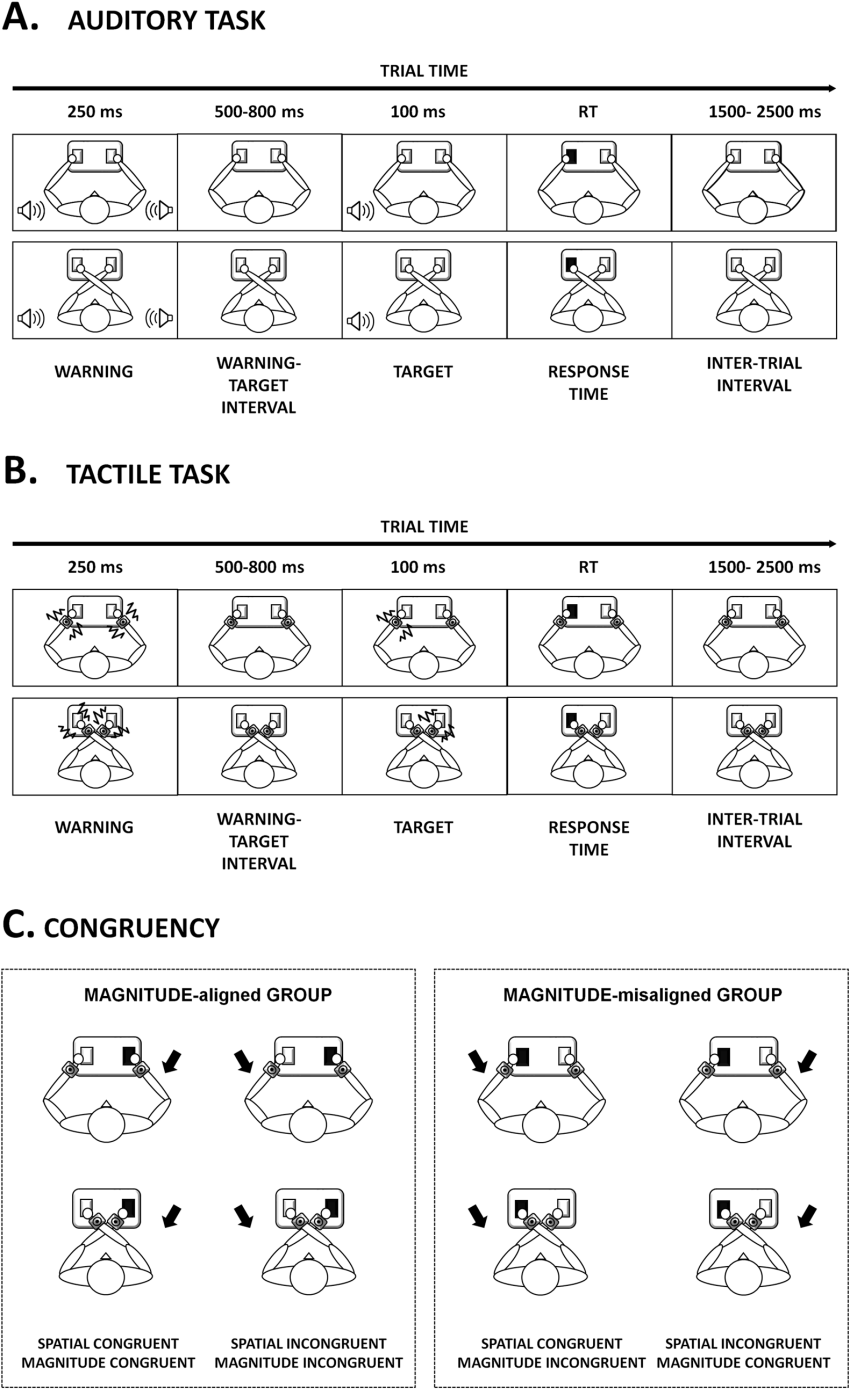
Schematic representation of task setup and procedure. Panel **A** and **B** represent the time window of the trial in uncrossed-hands posture (top) and crossed-hands posture (bottom) of auditory (**A**) and tactile (**B**) tasks. Panel **C** represents the schema of our experimental conditions in the case of high-frequency stimulus in the two groups, the black arrow represents stimulus position, black response key, the correct response key

Participants sat in a silent room with their hands on the push-button panel. All participants performed two types of high and low-frequency discrimination tasks: auditory stimuli and tactile stimuli. Each participant was randomly assigned to one of two groups. In the MAGNITUDE-aligned (MA) group (*n* = 19, 11 females, age [mean ± SD]: 26.21 ± 5.1 years) the response required was congruent with the mental magnitude line (i.e., low frequencies were represented on the left-hand space and high frequencies on the right-hand space). Participants had to press the left key for the low-frequency stimulation and the right key for the high-frequency stimulation in this group, and thus spatial congruency was maintained. In the MAGNITUDE-misaligned (MM) group (*n* = 19, 12 females, age: 27.79 ± 7.6 years), the required response was opposite to the mental magnitude line, i.e., the participant had to press the left key for high-frequency stimulation and the right key for low-frequency stimulation (see Fig. 1B). Thus, for the MM group, the magnitude SRC was incongruent with the spatial SRC.

For the duration of the experimental session, they were instructed to look at a fixation point 95 cm away and aligned with their body midline. The trial began with a warning (auditory or tactile) signal, followed by a random delay between 500 and 800 ms. After this delay, the target appeared for 100 ms in the left or right hemispace. Participants were instructed to respond as quickly as possible to the frequency of the stimuli (high or low), by pressing the left or right button, as appropriate, regardless of stimulus location. Response times longer than 2.5 s were considered null. The next trial started with a 1.5–2.5 s delay following the preceding trial's response (Fig. 1A, B). Participants performed the task either with their arms in a parallel position (uncrossed hands) or with their hands crossed over the body midline (crossed hands). Each task consisted of four blocks of 60 trials, two blocks for each hand position. The order of hand position and sensory modality tested was balanced among participants.

### Data analysis

Spatial S–R congruency was defined according to the relationship between the correct response key (left/right) and the stimulus position. A trial was considered spatially congruent if the stimulus side matched that of the push button (left/right) associated with the correct response, regardless of the arm posture (crossed/uncrossed) (Fig. 1C).

For each participant, we excluded all responses (6.3 ± 2.7 average among participants) that were three median absolute deviations above or below the median, as a robust method to remove outliers (Leys et al. 2013). Accuracy was calculated as the percentage of correct responses and reaction time was the mean of correct answers' reaction times. We calculated an integrated measure of speed and accuracy to control for interindividual differences in response strategy (Kanai and Rees 2011). The linear integrated speed-accuracy score (LISAS) (Vandierendonck 2017) has been demonstrated to detect effects present in either speed or accuracy and is effective in signaling a larger number of effects than is detectable by speed or accuracy data alone (Bollini et al. 2020; Vandierendonck 2018). LISAS is calculated as

1 $$ \begin{array}{*{20}c}    {{\text{LISAS}} = {\text{RT}}_{{{\text{cond}}}}  + {\text{PE}}_{{{\text{cond}}}}  \times \frac{{\sigma {\text{RT}}_{{{\text{tot}}}} }}{{\sigma {\text{PE}}_{{{\text{tot}}}} }}}  \\   \end{array}  $$

where RT_cond_ is the participant's mean response time (RT) for a condition, PE_cond_ is the participant's proportion of errors (PE) for the same condition, *σ*RT_tot_ is the participant's overall RT standard deviation for all conditions, and *σ*PE_tot_ is the participant's overall PE standard deviation for all conditions. In this way, errors are weighted by the RT and PE standard deviations ratio, and a similar weighting of the two components (RT and PE) is achieved (Vandierendonck 2018). Lower scores on LISAS indicate better performance (i.e., faster and more accurate), and vice-versa.

To test for differences between space and magnitude across the senses, we fitted a linear mixed-effects model to LISAS, using effects coding to describe the factor levels (Davis 2021) and restricted-maximum-likelihood (REML) as convergence criteria. The model's fixed effects were the between-subjects factor “Group” (MA and MM), the within-subjects factor “Hands Posture” (uncrossed and crossed), the within-subjects factor “Spatial congruency” (congruent and incongruent); the within-subjects factor “Sense” (auditory and tactile); and all the respective interactions. We modeled the within-subject effects as random intercepts nested within the subject. The formula describing our model in Wilkinson's notation (Wilkinson and Rogers 1973) is:

2 $$\begin{array}{c}LISAS \sim Group*Hands Posture*Congruency*Sense+\left(1|participant\right)+\\ \left(1|Hands Posture:participant\right)+\left(1|Congruency:participant\right) +\left(1|Sense:participant\right)\\ \end{array}$$

We performed *t*-tests on the model estimates using Kenward-Roger's degrees of freedom approximation (Luke 2017). Post-hoc tests were performed on the highest significant interaction estimate levels with Bonferroni correction for multiple comparisons. The same analyses were also repeated for reaction times and accuracy scores.

We ran an additional set of contrasts to test the SRC effect directly, or Δ-Spatial (i.e., Incongruent—Congruent trials) effect, for each combination of Group, Sense and Hands Posture levels. Analyses were done in R (R Core Team 2019). Model fitting was done using the package *lme4* (Bates et al. 2007), *t*-tests on the fixed-effects were done using the package *lmerTest* (Kuznetsova et al. 2017); and the post-hoc comparisons and the planned contrasts using the package *emmeans* (Lenth et al. 2020). The effect size (semi-partial *R*^2^, *R*_*p*_^2^) of each linear mixed model factor was calculated using the Kenward-Roger approach and package *r2glmm* (Jaeger et al. 2017).

## Results

**Fig. 2:**
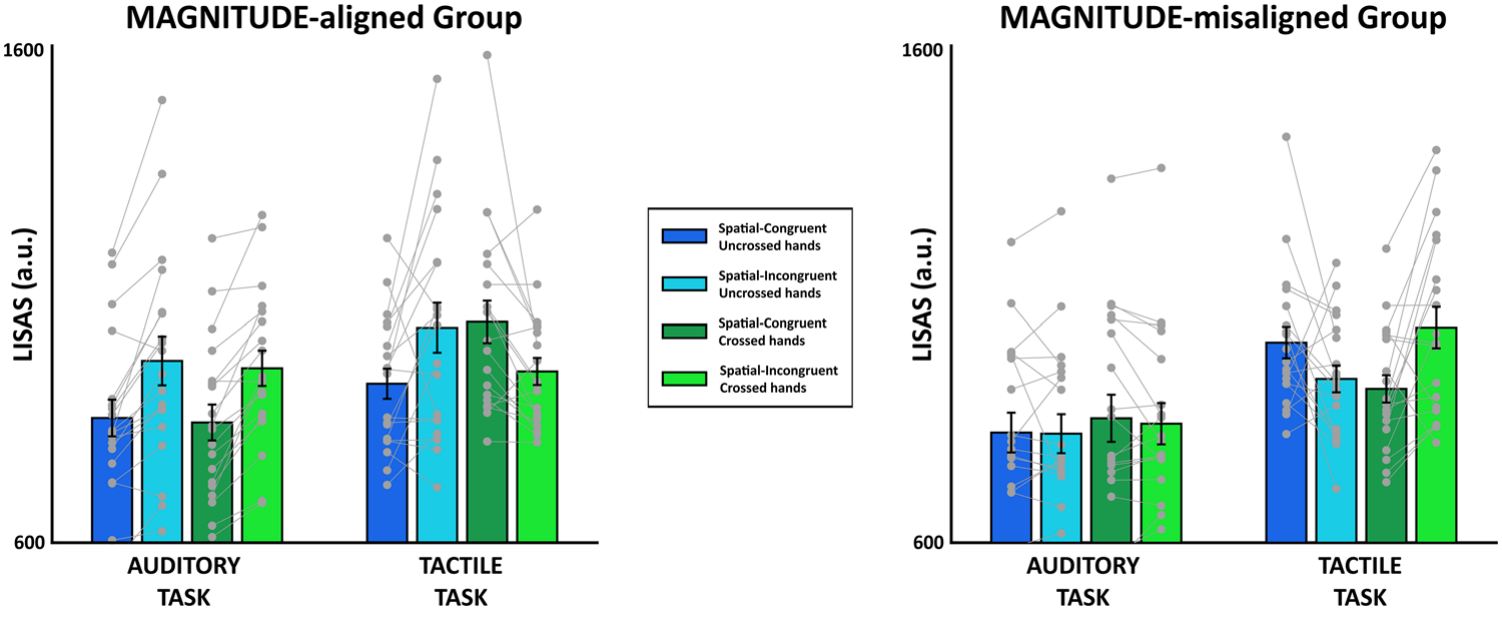
Results of MAGNITUDE-aligned and MAGNITUDE-misaligned groups in auditory and tactile stimulus–response tasks. Left panel: LISAS scores for the MAGNITUDE-aligned (MA) group. Right panel: LISAS scores for the MAGNITUDE-misaligned (MM) group. Error bars represent the standard error of the mean (SEM); gray points single-subject values

The linear mixed-effect model revealed a significant main effect for spatial-congruency (*t*(36) = − 3.46, *p* = 0.001, *R*_*p*_^*2*^ = 0.25) and sense (*t*(36) = − 4.66, *p* < 0.001, *R*_*p*_^*2*^ = 0.38) (Fig. 2). Moreover, the fixed-effects estimates of LISAS revealed a significant interaction between group and spatial-congruency (*t*(36) = 2.53, *p* = 0.016, *R*_*p*_^*2*^ = 0.15); among group, spatial-congruency and hands posture (*t*(144) = 5.12, *p* < 0.001, *R*_*p*_^*2*^ = 0.15); among group, spatial-congruency and sense (*t*(144) = 3.47, *p* < 0.001, *R*_*p*_^*2*^ = 0.08); and among group, spatial-congruency, hands posture and sense (*t*(144) = − 5.20, *p* < 0.001, *R*_*p*_^*2*^ = 0.16). For brevity only the four factor interaction will be fully discussed.

Post-hoc analysis revealed an SRC effect (i.e., congruent < incongruent) was present in both the MA and MM groups (Fig. 2), so participant showed lower LISAS values for spatial-congruent trials and higher values for spatial-incongruent trials. In the auditory task, group MA had a significant SRC effect for both hands posture levels (uncrossed: *t*(177) = 4.11, *p*_bonf_ < 0.001, Cohen's *d* = 1.33; crossed: *t*(177) = 3.9, *p*_bonf_ = 0.001, Cohen's *d* = 1.27). In the tactile task, group MA had a significant SRC effect only for the uncrossed hands posture treatment (*t*(177) = 4.02, *p*_bonf_ < 0.001, Cohen's *d* = 1.30), and a reverse SRC effect was present with crossed hands (*t*(177) = − 3.55, *p*_bonf_ = 0.004, Cohen's *d* = − 1.15). For the MM group in the auditory task, spatially-congruent and incongruent conditions did not differ in either hands posture groups (uncrossed: *t*(177) = − 0.08, *p*_bonf_ = 1, Cohen's *d* = − 0.03; crossed: *t*(177) = − 0.39, *p*_bonf_ = 1, Cohen's *d* = − 0.13). In the tactile task, group MM results were opposite those for group MA: a reverse SRC (i.e., congruent > incongruent) effect was present with uncrossed hands (*t*(177) = − 3.55, *p*_bonf_ = 0.004, Cohen's *d* = − 0.85), so lower LISAS values for spatial-incongruent trials and higher values for spatial-congruent trials. A significant SRC effect occurred for the crossed hands treatment (*t*(177) = − 3.55, *p*_bonf_ = 0.004, Cohen's *d* = 1.43). The results of reaction times and accuracy are reported in Supplementary Materials.

**Fig. 3:**
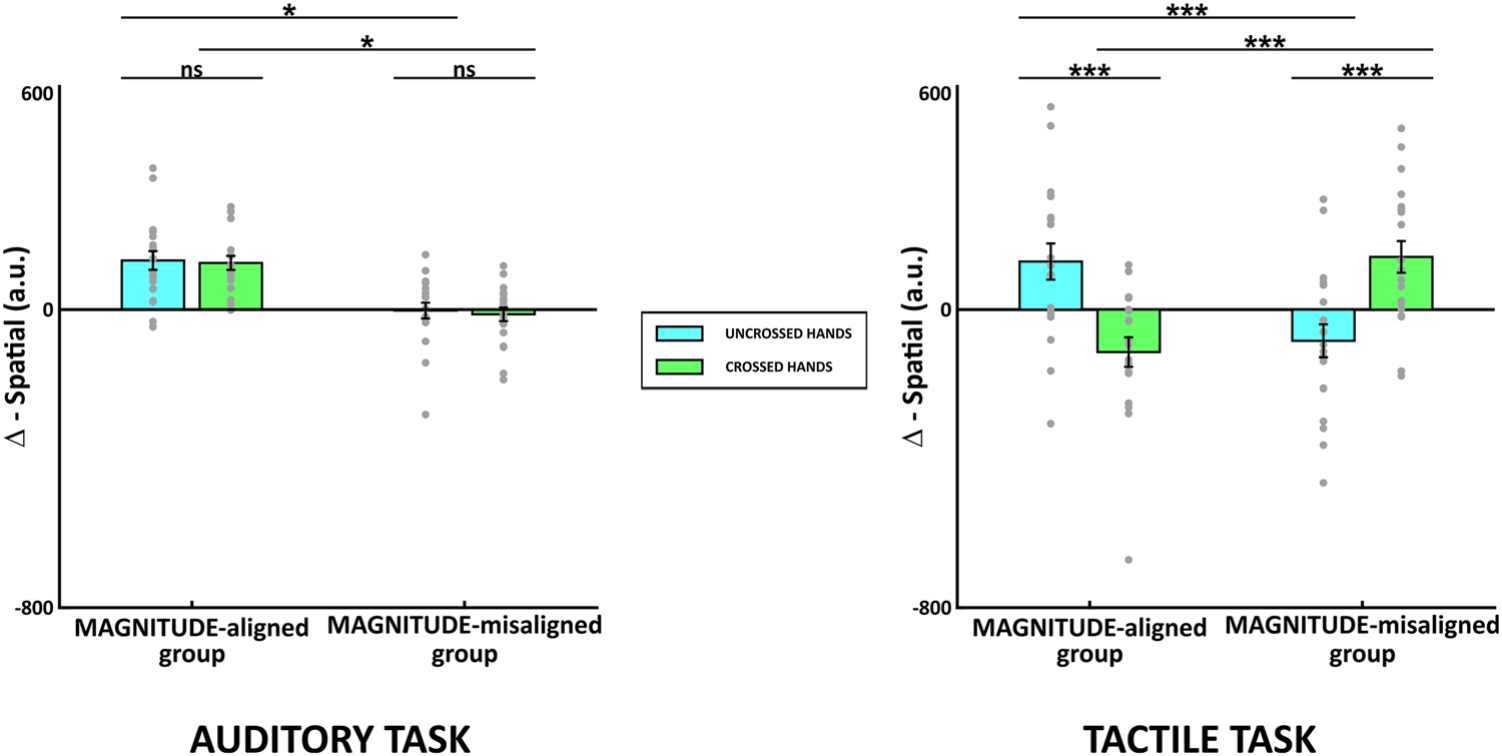
Δ-Spatial (spatial-incongruent MINUS spatial-congruent) for MAGNITUDE-aligned and MAGNITUDE-misaligned groups. The first panel on the left represents results for the auditory task. The panel on the right represents results for the tactile task. Error bars represent the standard error of the mean (SEM), gray points single-subject performance,* indicates p_bonf_<0.05, *** indicates p_bonf_ <0.001, ns indicates not significant result.

Using planned contrasts on Δ-Spatial we found that in the auditory task the two groups differ within each hands posture condition (uncrossed: *t*(177) = − 2.96, *p*_bonf_ = 0.014, Cohen's *d* = − 1.36; crossed: *t*(177) = − 3.04, *p*_bonf_ = 0.011, Cohen's *d* = − 1.39) (Fig. 3a), namely there were the difference between congruent and incongruent trials were significantly different from 0. However, the crossed versus uncrossed hands posture in the auditory task did not differ significantly within either group (MA: *t*(144) = 0.14, *p*_bonf_ = 1, Cohen's *d* = 0.07; MM: *t*(166) = 1.69, *p*_bonf_ = 0.37, Cohen's *d* = 0.95) (Fig. 3a), that is the difference between congruent and incongruent trials where not affected by hands posture. As with the auditory task, in the planned contrasts on Δ-Spatial for the tactile task, the two groups differ within each hands posture conditions (uncrossed: *t*(177) = − 4.69, *p*_bonf_ < 0.001, Cohen's *d* = − 2.15; crossed: *t*(177) = 5.63, *p*_bonf_ < 0.001, Cohen's *d* = 2.58) but the direction of the effect is opposite in the crossed hands group (Fig. 3b). In contrast to the auditory task, for the tactile task, Δ-Spatial differed significantly between uncrossed and crossed posture tasks within each groups (MA: *t*(144) = 5.35, *p*_bonf_ < 0.001, Cohen's *d* = 2.46; MM: *t*(144) = − 4.96, *p*_bonf_ < 0.001, Cohen's *d* = − 2.28; Fig. 3b), meaning that the hands posture affect the relationship between congruent and incongruent trials.

## Discussion

We hypothesized that the relationship between magnitude and spatial representation is processed differently in different sensory modalities. In agreement with our hypothesis, we found that the balance between space and magnitude differs in auditory and tactile SRC tasks. Moreover, we found that the spatial frame activated during the tasks is elicited by the sensory inputs, regardless of the interaction between task and magnitude.

### Effect of magnitude for audition

In the auditory modality, the main result was that, in the MAGNITUDE-misaligned group, participants showed neither spatial congruency nor magnitude congruency effects. The magnitude can explain this result and spatial effects canceling each other as if they had the same size but opposite directions. Indeed, as demonstrated from the MAGNITUDE-aligned group, at least one SRC effect should be present, for the group showed faster and more accurate responses when the stimulus was congruent in both magnitude and space. We must emphasize that the SPARC effect, i.e., the SRC effect with high-low frequency sounds, is believed to be mapped mainly with a vertical representation, so it emerges with a vertical response mapping code (Cho et al. 2012; Nishimura and Yokosawa 2009; Rusconi et al. 2006). Magnitude is represented in the horizontal space in the auditory modality when participants are asked to judge magnitude, while when the pitch height is irrelevant, a horizontal SMARC appears only in musicians (Cho et al. 2012). Therefore, in our auditory task, in the case of musicians or vertical response mapping, the magnitude may overcome spatial representation, as in the tactile task performance in the MAGNITUDE-misaligned group.

### Effect of magnitude for touch

On the contrary, in the tactile modality, participants used magnitude over spatial representation. In other words, they showed the Spatial-Tactile Association of Response Code (STARC) effect: for tactile inputs, low quantities were represented in the left space and high quantities in the right space (Bollini et al. 2020). Indeed, our results showed a reverse spatial SRC effect (also known as reverse Simon effect) resulting from the STARC effect's interference.

### Sensory modality vs. magnitude and space

This study demonstrates that the sensory modality modulates the relationship between magnitude and space representation. Indeed, participants weighted tactile and auditory compatibility in magnitude and space differently, at least in the horizontal space; a stronger effect for magnitude over spatial congruency was observed for the tactile modality, while in the auditory modality, no effect was observed. This happened because both magnitude and spatial congruencies had the same weight, but the S–R compatibilities had opposing directions, leading to the nullification of any effects. This result led us to conclude that in the auditory modality, magnitude and spatial representation are equally represented, while in the tactile modality, the magnitude has a larger effect than spatial representation. It has been hypothesized that magnitude, space, time and size share a universal common representation in the parietal cortex (Bueti and Walsh 2009). Specifically, it has been proposed that parietal cortex activation reflects the convergence of different sensory inputs, each with its specialized representation, leading to a magnitude estimation that not only depends on the input itself but is also affected by context (Petzschner et al. 2015). In this view, the task designed to discriminate between MAGNITUDE-aligned and MAGNITUDE-misaligned scenarios worked as a universal cognitive context, which was added to the spatial encoding of touch and hearing the sensory inputs.

Our findings reveal the presence of a map that translates magnitude, a non-spatial domain, to a mental spatial continuum (from left-to-right) that interacts directly with the spatial representation for both the auditory and tactile modalities. However, we found that the magnitude of the spatial representation's effect differs between the two modalities in the same participants.

### Reference of frames in the space-magnitude relationship

Our study's second aim was to investigate the reference frames in the space-magnitude relationship. We created a conflict between internal and external coordinates to disentangle the reference frames by instructing our participants to respond with their hands crossed over the body midline. In this condition, we created a misalignment between the stimuli's spatial position and the response effectors, i.e., the hands. As demonstrated by the significant interactions with the hands' posture factor and the subsequent post-hoc tests, auditory and tactile tasks in both groups evoked a different spatial reference frame. Indeed, when hands were crossed in the tactile modality, the effect's direction was always opposite to the congruency effect in the uncrossed hands' condition. The congruency effect was always in the same direction in the auditory modality, regardless of hands postures. These findings can be interpreted as indicative of the dominance of the external frame of reference for the auditory task; crossing hands did not affect performance. In the tactile task, however, participants relied on an internal frame of reference, and thus the congruency effect was reversed in the hands crossed posture, following body-centered coordinates. The crossed hands condition has been widely used to test the spatial coordinates during S–R congruency tasks using different sensory inputs, including visual inputs (Phillips and Ward 2002; Roswarski and Proctor 2000; Ruzzoli and Soto-Faraco 2017; Wallace 1971; Wascher et al. 2001), auditory inputs (Crollen et al. 2017; Roder et al. 2007; Roswarski and Proctor 2000) and tactile inputs (Bollini et al. 2020; Medina et al. 2014; Ruzzoli and Soto-Faraco 2017). These studies demonstrate that vision and hearing rely on the allocentric frame while touch relies on the egocentric frame. However, which reference frame is involved with magnitude remains an open question. In recent studies (Mourad and Leth-Steensen 2017; Viarouge et al. 2014), the presence of a hierarchical spatial frame that may activate internal or external coordinates based on the experimental context has been proposed. Our results are in line with these studies. We found that the spatial reference frame seemed to change for both magnitude and spatial S–R compatibility effects depending on the sensory modality used.

## Conclusions

In conclusion, we demonstrate here that sensory modalities play an important role in the representations of space and magnitude. Our manipulation revealed that auditory and tactile sensory channels balance space and magnitude differently. While magnitude prevails over space in the tactile task, space and magnitude appear to be balanced in the auditory task. Lastly, we demonstrate that the spatial reference frame activated during tasks follows the coordinates elicited by the sensory inputs, namely an egocentric spatial frame for the tactile task and an allocentric spatial frame for the auditory task, regardless of the congruency between space and magnitude.

## Supplementary Information

Below is the link to the electronic supplementary material.

Supplementary file1 (DOCX 19 KB)

## Data Availability

The authors' raw data supporting the conclusions of this article will be made available without undue reservation.
