# Supplementary material for: Different mechanisms of magnitude and spatial representation for tactile and auditory modalities
Source: Exp Brain Res. 2021 Aug 20;239(10):3123–32. doi: 10.1007/s00221-021-06196-4 (PMC8536643; doi:10.1007/s00221-021-06196-4)
Supplement: Supplementary file 1 — Supplementary file1 (DOCX 19 KB) [file 221_2021_6196_MOESM1_ESM.docx]

**Supplementary Materials:**

**Different mechanisms of magnitude and spatial representation for tactile and auditory modalities.**

*Experimental Brain Research*

Alice Bollini^1^*, Davide Esposito^1-2^, Claudio Campus^1^, Monica Gori^1^

^1^ Unit for Visually Impaired People, Istituto Italiano di Tecnologia, Genoa, Italy.

^2^ DIBRIS, Università di Genova, Genoa, Italy.

*Corrisponding author. Contact: [alice.bollini@iit.it](mailto:alice.bollini@iit.it)

- Reaction time analysis

The reaction time model outcome revealed a significant main effect for spatial-congruency, spatial-congruency (*t*(36) = -4.14, *p* < 0.001, *R_p_^2^* = 0.32) and sense (*t*(36) = -4.64, *p* <0.001, *R_p_^2^* = 0.37). Moreover, the fixed-effects estimates of RT revealed a significant interaction between group and spatial-congruency (*t*(36) = 2.17, *p* = 0.046, *R_p_^2^* = 0.12); among group, spatial-congruency and hands posture (*t*(144) = 2.64, *p* = 0.009, *R_p_^2^* = 0.15); and among group, spatial-congruency, hands posture and sense we obtained a near-significant interaction (*t*(144) = -1.97, *p* = 0.050, *R_p_^2^* = 0.026).

- Accuracy analysis

The linear mixed-effect model on accuracy scores revealed a significant main effect for spatial-congruency (*t*(36) = 2.72, *p* = 0.01, *R_p_^2^* = 0.17) and sense (*t*(36) = 2.94, *p* =0.006, *R_p_^2^* = 0.19). Moreover, the fixed-effects estimates of accuracy scores revealed a significant interaction between group and spatial-congruency (*t*(36) = -2.90, *p* = 0.006, *R_p_^2^* = 0.19); among spatial-congruency and sense (*t*(144) = 1.99, *p* =0.049, *R_p_^2^* = 0.027); among group, spatial-congruency and hands posture (*t*(144) = -5.91, *p* <0.001, *R_p_^2^* = 0.195); among group, spatial-congruency and sense (*t*(144) = -3.96, *p* <0.001, *R_p_^2^* = 0.098); and among group, spatial-congruency, hands posture and sense (*t*(144) = 6.56, *p* <0.001, *R_p_^2^* = 0.23). For brevity only the four-factor interaction will be fully discussed.

Post-hoc analysis revealed an SRC effect (i.e., congruent > incongruent) was present in both the MA and MM groups, so participant showed higher accuracy values for congruent trials and lower values for incongruent trials. In the auditory task, group MA had a significant difference between spatial-congruent and incongruent accuracy scores for both hands posture levels (uncrossed: *t*(176) = -4.11, *p_bon_*_f_ <0.001, Cohen's *d* = -1.35; crossed: *t*(176) = -4.14, *p_bonf_* <0.001, Cohen's *d* = -1.36). In the tactile task, group MA had a significant SRC effect for the uncrossed hands posture treatment (*t*(176) = -4.82, *p_bonf_* <0.001, Cohen's *d* =-1.58), and a reverse SRC effect (i.e., congruent > incongruent) was present with crossed hands (*t*(176) = 4.90, *p_bonf_* < 0.001, Cohen's *d* = 1.60). For the MM group in the auditory task, spatially-congruent and incongruent conditions did not differ in either hands posture groups (uncrossed: *t*(176) = 0.32, *p_bonf_* = 1, Cohen's *d* = 0-11; crossed: *t*(176) = 1.2, *p_bonf_* = 1, Cohen's *d* = 0.39). In the tactile task, group MM results were opposite those for group MA: a reverse SRC effect was present with uncrossed hands (t(176) = 3.25, p_bonf_ = 0.011, Cohen's d = 1.06), so higher accuracy values for incongruent trials and lower values for congruent trials. A significant SRC effect occurred for the crossed hands treatment (*t*(176) = -4.50, *p_bonf_* < 0.001, Cohen's *d* = -1.48).
